# Soil carbon fluxes and balances of crop rotations under long-term no-till

**DOI:** 10.1186/s13021-020-00154-3

**Published:** 2020-09-16

**Authors:** João Paulo Gonsiorkiewicz Rigon, Juliano Carlos Calonego

**Affiliations:** College of Agricultural Sciences-Department of Crop Science, São Paulo State University (UNESP), Lageado Experimental Farm, Botucatu, SP Brazil

**Keywords:** Cover crop, Cropping system, Crop straw, C and N inputs

## Abstract

**Background:**

A field study with the same crop rotations was conducted to test the hypothesis that the soil Carbon fluxes and balances could vary according to the crop species and also mitigate carbon dioxide (CO_2_) emission. This study aimed to assess the CO_2_ emission from crop rotations according to C and N inputs from crop residue, the influences on soil organic carbon (SOC) and total soil nitrogen (TN) stocks, identifying the soybean production systems with positive C balance. Triticale (*x Triticosecale*) or sunflower (*Helianthus annuus*) are grown in the fall/winter; sunn hemp (*Crotalaria juncea*), forage sorghum (*Sorghum bicolor*), pearl millet (*Pennisetum glaucum*), or fallow are the spring treatments, and soybean as a main crop in summer.

**Results:**

We found that high C inputs from crop residues modify the C dynamics in crop rotations by reducing the C output (CO_2_) and increasing C sequestration in the soil. In general, the higher SOC, C stocks, and TN in soil surface were due to higher C and N inputs from sunn hemp or forage sorghum crop residues in spring. These crops also produced lower accumulated CO_2_ emissions and, when rotating with triticale in the fall-winter season resulted in a positive C balance, making these soybean crop rotations more efficient.

**Conclusion:**

Our study suggests the ideal crop species choice in a rotation can mitigate the CO_2_ emissions by increasing C and N input from crop residues and consequently SOC and C stocks. In particular, crop rotation comprises an important tool to achieve a positive C balance, mitigate CO_2_ emissions and provide an additional ecosystem service to soybean cultivation option.

## Background

Soil comprises one of the largest reserves of Carbon (C) in the biosphere, and depending on the soil management used, it can be considered an important sink and act directly in reducing carbon dioxide (CO_2_) emissions to the atmosphere [1], mitigating the impact of current and future climate change [2–4].

In Brazil, the agricultural sector is currently the largest source of global greenhouse gas (GHG) emissions, with a 34% share [5]. However, the potential for agricultural mitigation is often ignored [6]. Thus, soil conservation management such as no-till and crop rotations, mainly adding cover crops, are some strategies when combined can increase soil organic carbon (SOC) [7], nutrient cycling and mitigate GHG emission in agricultural systems [8–10].

Carbon dioxide emissions, as well as SOC increases, also depend on the crop rotations used in the agricultural system, which are affected by the quality of crop residue left on the soil and the amount of easily mineralizable C [11, 12]. However, it varies according to weather conditions, soil texture [13], crop rotation species [14]. Recognizing this complexity, recent studies have included crop rotations as a factor that directly affects soil CO_2_ emissions [14–16].

Quality parameters of crop residues such as nitrogen (N) content, lignin and polyphenols could alter the dynamics of soil C and N, and microbial activity [17, 18], and the rate of mineralization and consequently CO_2_ emitted to the atmosphere [14].

In general, legumes as cover crops increase N input, and consequently, the total soil nitrogen (TN) on the soil surface and could be considered a key factor in C sequestration in tropical soils [7, 19]. Moreover, grasses have higher biomass production and levels of recalcitrant compounds in crop residues, allowing slower mineralization covering the soil surface [20, 21]. Such characteristics affect CO_2_ emissions and soil C sequestration depending on crop rotation species.

Knowledge of the relationship between crop residue input and C cycle dynamics related to crop rotation species is still lacking, particularly under no-till. To fill this gap in the literature on the dynamics of the carbon cycle, the goal of this study was to determine CO_2_ emissions from crop rotations according to C and N inputs from crop residue, the influence on SOC and TN stocks, identifying the soybean production systems with positive C balance. This allowed for testing several specific hypotheses: (i) the C and N inputs from crop rotation impact positively on SOC and C stocks; (ii) a legume as a cover crop offset the CO_2_ emissions by a positive C balance from soybean cropping systems; (iii) higher C inputs of crop rotations sequester C and mitigate CO_2_ emission.

## Materials and methods

### Site description

**Fig. 1:**
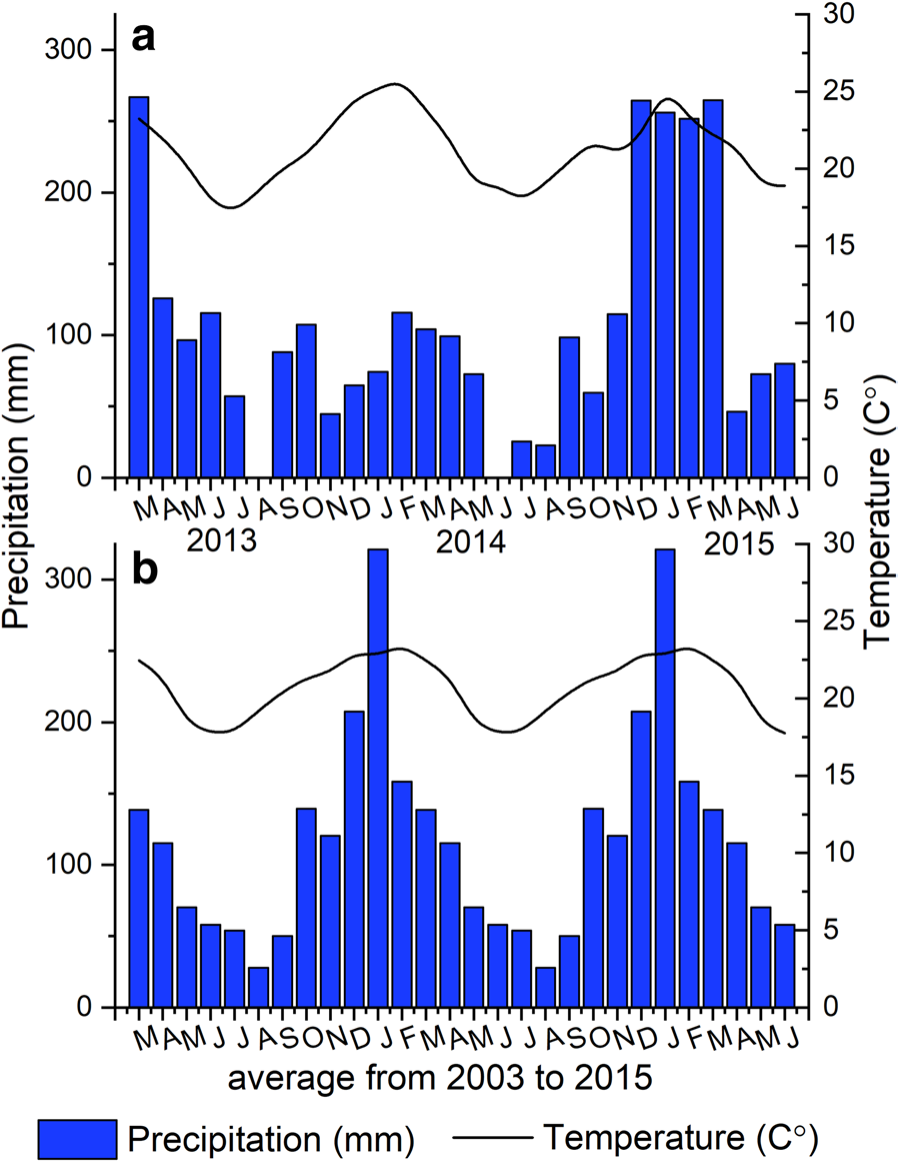
Accumulated precipitation, and mean monthly temperature between March 2013 and June 2015 (**a**), and March 2003 to June 2015 (**b**)

The field experiment was conducted in Botucatu, SP, Brazil (22°49′ S;48°25′ W at an altitude of 780 m), on a Typic Rhodudalf (Soil Survey Staff, 2014). The climate is mesothermal with a dry austral winter and a well-defined dry season from May to September, with mean annual rainfall of 1450 mm (Fig. 1). The soil chemical [22] and physical [23] characteristics are shown in Table 1.

**Table 1:** Selected chemical, physical and granulometric properties of the soil in August 2013

Soil attribute	Soil profile (m)			
	0–0.1	0.1–0.2	0.2–0.4	0.4–0.6
pH^a^	5.1	4.5	4.5	4.5
Al (mmol_c_ dm^−3^)	0.2	0.7	0.6	0.8
Ca^b^ (mmol_c_ dm^−3^)	41	19	20	21
Mg^b^ (mmol_c_ dm^−3^)	33	13	13	12
K^b^ (mmol_c_ dm^−3^)	1.9	1.5	0.6	0.6
P^b^ (mg dm^−3^)	33	17	9	8
Sand^c^ (g kg^−1^)	108	100	84	66
Silt^c^ (g kg^−1^)	237	245	211	204
Clay^c^ (g kg^−1^)	655	655	705	730
Microporosity (m^3^m^−3^)	0.42	0.41	0.39	0.41
Macroporosity (m^3^m^−3^)	0.12	0.09	0.09	0.09
Bulk density (Mg m^−3^)	1.32	1.37	1.29	1.26

^a^pH determined in CaCl_2_ 0.01 mmol L^−1^

^b^Available P and Ca^2+^, Mg^2+^, K^+^ were extracted using a cation and anion exchange resin

^c^Clay, silt and sand contents determined by pipet method [24]

### Experimental design

The experiment was laid out as a split-plot in a randomized complete block design with eight treatments and four replications. The main plots consisted of species cultivated in the fall and winter, and subplots of crops grown in spring for green manure. The experiment began in 2003 with triticale [*X Triticosecale* (Wittmack)] or sunflower [*Helianthus annuus* (L.)] grown in fall-winter in 32 m × 5 m plots, followed by pearl millet [*Pennisetum glaucum* (L.)], sunn hemp [*Crotalaria juncea* (L.)], and sorghum [*Sorghum bicolor* (L.)] or fallow soil during the spring, in 8 m × 5 m subplots, with soybean grown [*Glycine max* (L.) Merrill] over the entire area in the summer (Additional file 1: Table S1). The species cultivated in the fall-winter, spring and summer seasons were repeated annually from 2003 to the 2015 season.

### Crop rotations management since 2003 to 2015

#### Fall-winter crops

The sunflower and triticale were sown on April annually under row spacing of 0.34 m and 0.17 m, and seeding rate of 8 and 165 kg ha^−1^, respectively, to sunflower and triticale. No fertilizers were used in winter crops since 2003 to 2015. Grain harvest was carried out on ending of Augusto or initial of September, and on the next days, the chemical management of the cultural remains was carried out.

#### Spring cover crop seasons

After fall-winter harvest, the following spring cover crops were sown, around second half of September, except for fallow treatment, at 0.17 m spacing, with 15, 25 and 30 kg ha^−1^ rate of seeds of forage sorghum, pearl millet, and sunn hemp, respectively. The fallow plots were chisel plowed in 2003, 2009, and 2013 before soybean planting. Also, no fertilizers were used in spring crops. At the pre-flowering stage, (end of November or beginning of December), cover crops were chemically desiccated with glyphosate, and residues were left on the soil surface.

#### Soybean crop seasons

The No-till sowing of soybean was performed in end of November or beginning of December under row spacing of 0.45 m. The soybean cultivars varied since 2003 to 2015, and the population was targeting according to recommendations, and fertilized with 50 kg ha^−1^ of K_2_O and 50 kg ha^−1^ of P_2_O_5_ as potassium chloride and triple superphosphate respectively, being the same fertilizers rate used since 2003 to 2015. Desiccation were chosen to accelerate harvesting in R7.3. The soybean harvest were carried out normally on April, with three 5 m long central lines of each subplot being used with a plot harvester. Soybean yield was calculated by correcting grain yield moisture to 13%.

### Sampling

#### C and N inputs of crop residues

Two crop residue samples (0.25 m^2^ each) were randomly collected in each subplot after the respective crop management: (i) spring crops 2013; (ii) soybean 2013/2014; (iii) fall-winter 2014; (iv) spring crops 2014; (v) soybean 2014/2015. The samples were dried at 60 °C for 48 h and weighed. Then the samples were ground, homogenized, and a subsample was used to determine the biochemical characteristics (van Soest and Wine, 1968), determined in an Ankom 220 fiber analyser (acid detergent fibre). The other part of the samples was used for analyzing C and N crop residue contents, as well as soybean and triticale grain C content, by elemental analyzer (LECO-TruSpec^®^ CHNS).

#### SOC and TN

Three soil subsamples (April, 2013) were sampled from each experimental unit at 0–0.1 m soil depth using a probe auger. The samples were air-dried, ball-milled, and SOC and TN content were determined in an elemental analyzer (LECO-TruSpec^®^ CHNS). Undisturbed soil samples were collected by the volumetric ring method [25]. The C and N stocks were calculated according to Eq. 1 [26]:

1 $$C\ or\;N\;stock = \frac{SOC\; or\;TN \times SD \times SL}{10}$$

where SOC is the soil organic carbon (g kg^−1^); TN is the total soil nitrogen (g kg^−1^); SD is the soil bulk density (Mg m^−3^); SL is the soil layer (10 cm).

#### Assessments of soil CO_2_ emission, soil temperature, and soil moisture

Polyvinyl chloride (PVC) collars (12 cm high and 20 cm wide) with thin-walled were installed in the crop rows, with the lower edge buried 5 cm in the soil where they remained until the respective crop management seasons. CO_2_ emissions were performed using a portable infrared gas analyzer (IRGA, LI-8100A, Li-Cor^®^), between 8:00 am and 11:00 am, configured with a reading of 120 s, with 15 s pre-purging and 15 s for post-purging, with automatic regression of CO_2_ flux in µmol m^−2^ s^−1^, transformed into g m^−2^ h^−1^. Soil temperature and volumetric moisture at 5 cm depth (Time Domain Reflectometry) 5TM^®^ (Decagon Devices) were determined together adjacent to the PVC collars. The determinations were performed at 1, 3, 8, 15, 30, 60, 90, and 120 d after sowing (DAS) of 2013/2014 soybean; fall-winter 2014 crops; and soybean of 2014/2015, and at 1, 3, 8, 15, and 30 DAS of spring crops 2014.

### Data analysis

Cumulative CO_2_ emissions were calculated by trapezoidal integration of daily emissions using Origin 8 software (OriginLab, Ltd., Northampton, MA, USA). Data were converted to carbon equivalent (C-CO_2_). The relative C-CO_2_ emission of soybean grain yield was calculated according to Eq. 2:

2 $$Relative\;emission = \frac{{soybean\;C - CO_{2} }}{soybean \;yield}$$

where: soybean C–CO_2_ is the cumulative emission during soybean season (t C–CO_2_ ha^−1^), soybean yield is the soybean grain yield (t ha^−1^), in kg C–CO_2_ kg^−1^. In each crop season, the C inputs from the roots of each crop rotation species were estimated based on the C of the crop residues [27]. The shoot: root ratios used to winter crops at harvest time were 2.8 to triticale and 5.1 to sunflower. For spring crops at the pre-flowering stage, the shoot: root ratios were 10.5, 14.2, and 4.4 respectively to sunn hemp, pearl millet, and forage sorghum. The C balance of each crop season was calculated according to Eq. 3 for the spring crops, and for Eq. 4, for the soybean seasons 2013/2014 and 2014/2015 and winter crops of 2014. The accumulated balance was obtained by summing the C balance of each crop for each treatment. The crop rotations under long-term NT influence soil organic C mainly at 0–0.1 m soil depth [7], and the C balance was calculated by accounting the 0.0–1 m soil depth for the C stocks in reference to the C fallow treatment stock by Eq. 5.

3 $$C\;balance = \frac{C\; input\; by\; crop\; residue + C\; input\; by\; previous\;crop*0.5 + C\;root}{{C - CO_{2} \;emission}}$$

4 $$C\;balance = \frac{C\;by\;crop\;residue + C\;input\;by\;previou\; crop*0.5 + C\;root + C\;in\;grain\;yield}{{C - CO_{2} \;\;emission}}$$

5 $${\text{Net}}\;{\text{C}}\;{\text{budget}} = Accumulated\;balance + C\;stock\; 2013 - C\;stock\;2013 \;fallow$$

where C input is from crop residues on soil surface of each crop (Kg ha^−1^) C root is the estimated cumulative C for each crop species (Kg C ha^−1^); C grain yield is the export of C of grains at harvesting (Kg C ha^−1^); C–CO_2_ is the accumulated emission in each crop. Homoscedasticity and normality of the data were tested using a randomized complete block design with four replicates. Data were subjected to analysis of variance (p < 0.05) by SAS version 9.2 [28], and the pairwise contrasts were performed using least significant differences (LSD, p < 0.05).

## Results

### C and N input by crop rotation

**Fig. 2:**
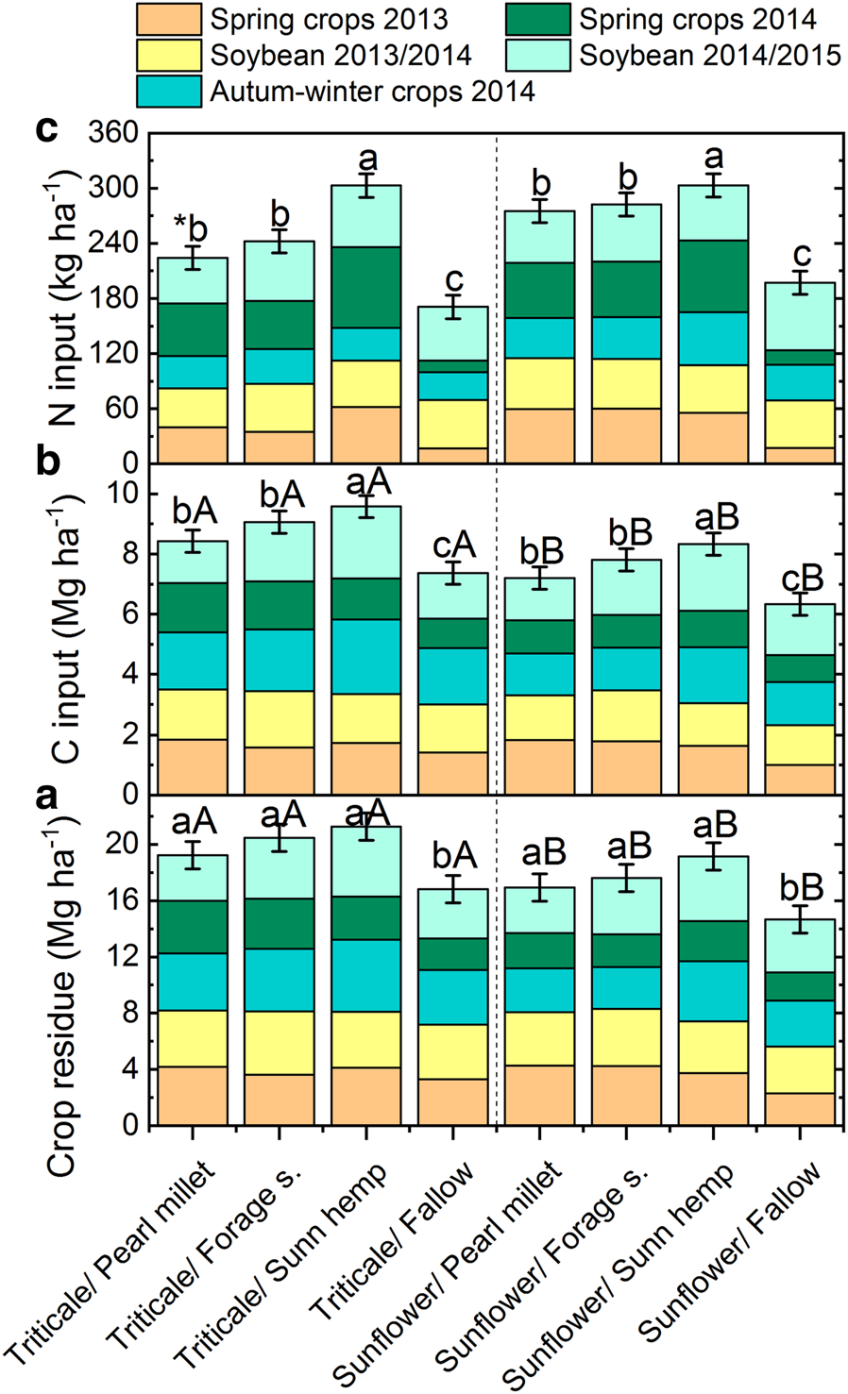
Amount of crop residue (**a**), Carbon (**b**), and Nitrogen (**c**) inputs from crop rotations in spring 2013, soybean 2013/2014, fall-winter 2014, spring 2014, and soybean 2014/2015. Error bars represent standard deviation of data. *Means followed by different letters, lower-case letters within spring treatments and upper-case letters within winter crops differ from each other by the paired *t* test (LSD, p < 0.05)

The crop residue characteristics on soil surface differed (p < 0.05) between fall-winter and mainly spring crops, but no interaction was observed between them. Across the five crop seasons analyzed, the cumulative amount of crop residue and C inputs on soil surface with triticale in fall-winter were, respectively, 2.34 and 1.2 t ha^−1^ higher (p < 0.05) than sunflower crop residue (Fig. 2a, b). In spring treatments, maintaining soil uncovered (fallow treatment), even for only about 60 days, the cumulative crop residue amount and the C and N inputs were noticeably lower (p < 0.05), compared to the treatments that had cover crops in the spring season. Regardless of spring species, crop rotations accumulated similar cumulative amounts of crop residue (p < 0.05; average 19.1 t ha^−1^). On the other hand, the accumulated contributions of C and N differed between species, ranging from 7.81 to 8.95 t C ha^−1^ and from 250 to 301 kg N ha^−1^, representing between a 15 to 30% increase in C input, and 35 to 63% in N input with the use of cover crops in the spring to the detriment of fallow treatment. Overall, across the seasons, the sunn hemp stood out with the highest N inputs (p < 0.05), independent of fall-winter crops (Fig. 2b, c).

**Fig. 3:**
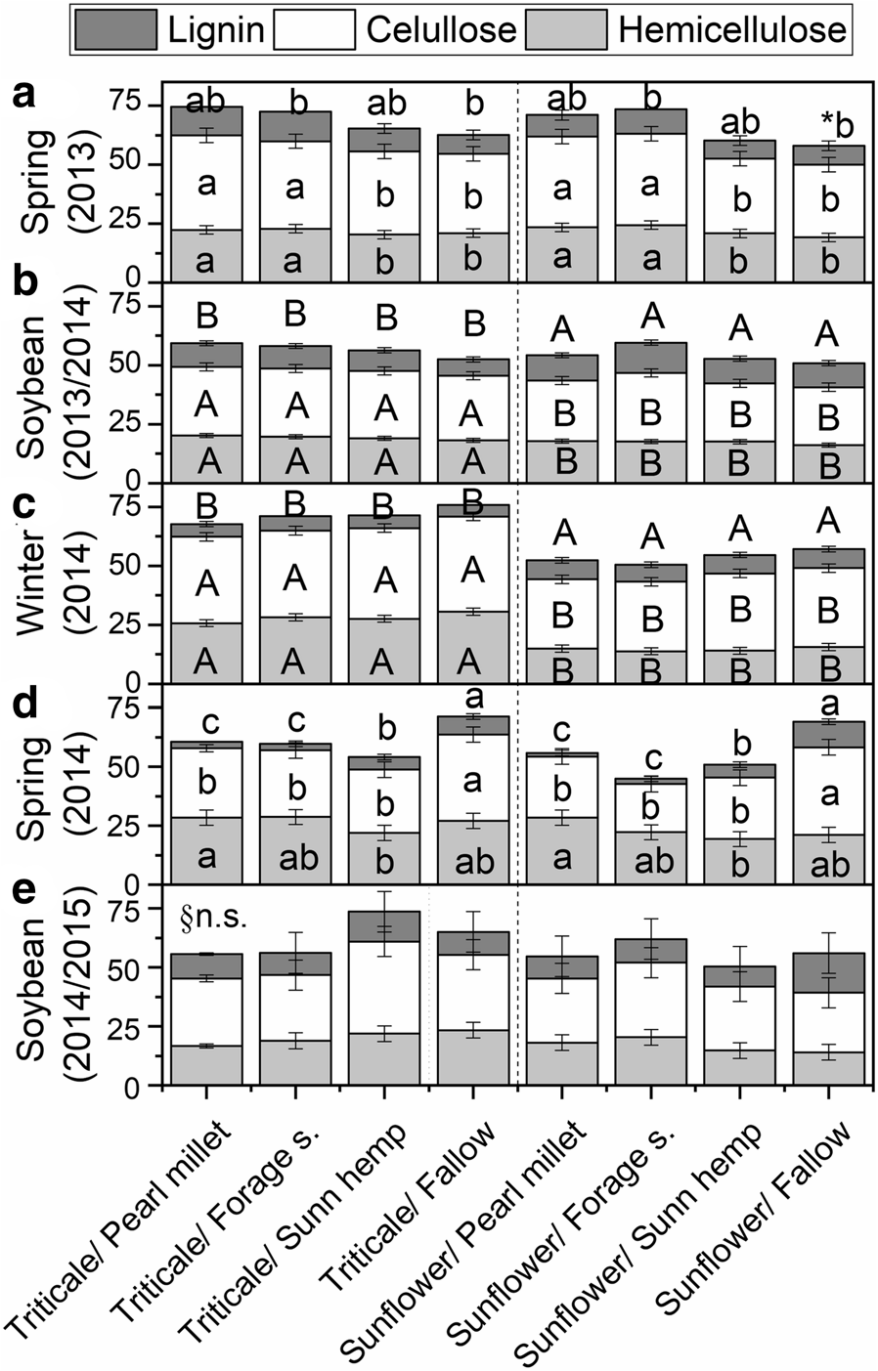
Hemicellulose, cellulose and lignin content (%) of crop residue according to crop rotations in spring 2013 (**a**), soybean 2013/2014 (**b**), fall-winter 2014 (**c**), spring 2014 (**d**), and soybean 2014/2015 (**e**). Error bars represent standard deviation of data *Means followed by different letters, lower-case letters within spring treatments and upper-case letters within winter crops differ from each other by the paired t-test (LSD, p < 0.05). ^§^n.s. no significative

Besides the amount of crop residue, the quality also differs between crop rotations. The quality of crop residue was impacted by spring treatments in spring seasons (Fig. 3a, d) and by winter crops in soybean 2013/2014 and winter crops (Fig. 3b, c). The lignin content from fallow treatment was higher, on average, from 18 to 23% for spring crop species, and a greater cellulose content in 2014 season, as well in 2013, no differing from sunn hemp. Concerning the winter crops, the hemicellulose, cellulose, and lignin contents were at least 24 and 14% higher, and 12% lower with triticale residue, respectively, compared to sunflower (Fig. 3b, c). Neither winter crop nor spring cover crop affected the crop residues in soybean 2014/2015.

Crop rotations under long-term NT were influenced mainly by spring treatments, the SOC, TN, and C stock at the soil surface (0–0.1 m) (Additional file 2: Table S2). Sunn hemp as cover crop in spring increase (p < 0.05) around 10 and 12% of SOC and C stock compared to fallow treatment. Similar behavior occurred between these treatments in TN contents and N stock, with values of 8% and 11% higher with the legume cropping compared to the fallow. In the soil profile analyzed (0–0.6 m, data no shown), C stocks were around 5 t ha^−1^ higher (p < 0.05) when triticale was cropped in fall-winter compared to sunflower (data not shown).

### CO_2_ emission

The climatic conditions during the experiment were typical of the region, with concentrated rainfall between spring and summer seasons (Fig. 1). Specifically between December 2013 and January 2014, the decrease in rainfall was associated with high temperatures (Fig. 1a), compared to the averages since the implementation of the experiment in 2003 (Fig. 1b).

**Fig. 4:**
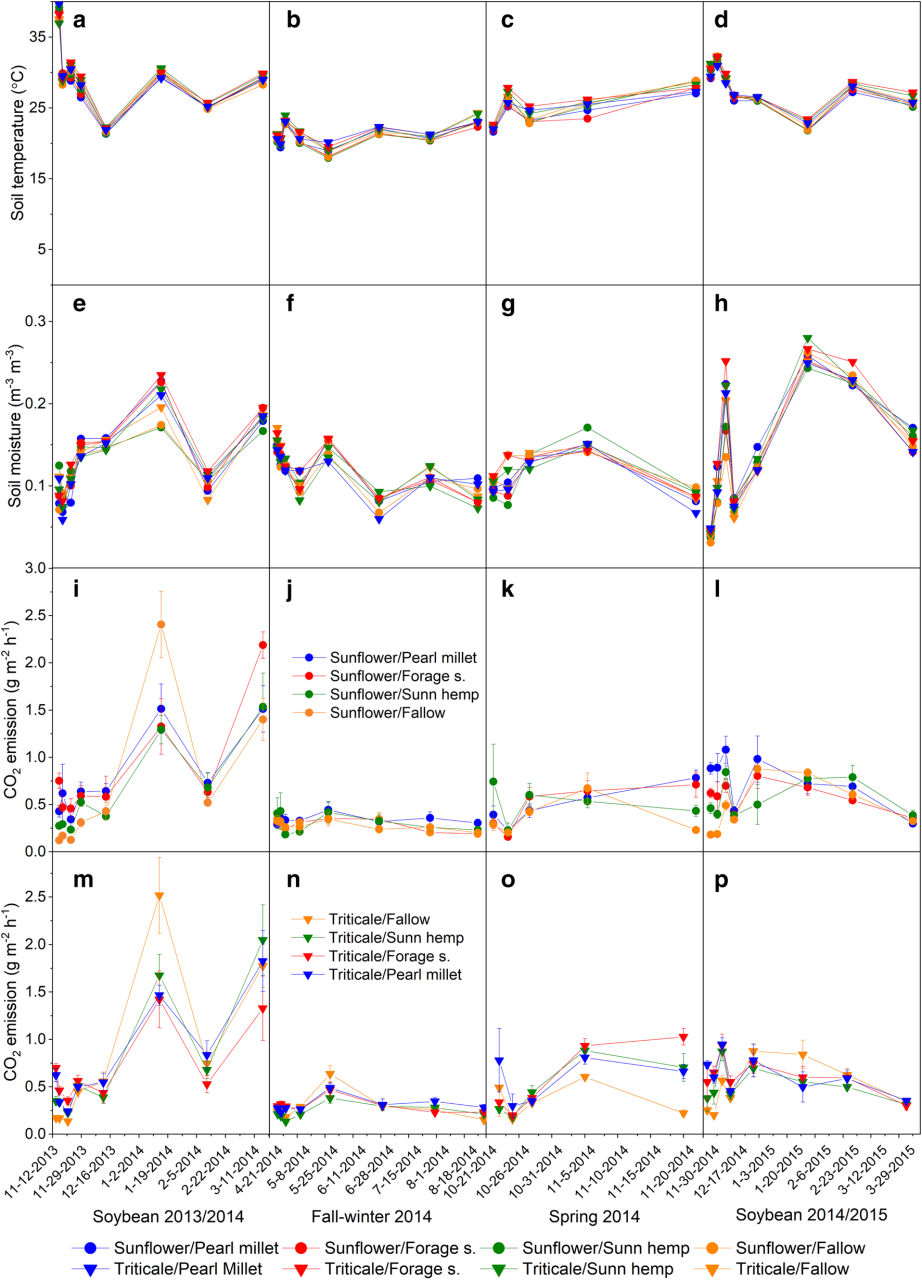
Soil temperature (**a**–**d**), moisture (**e**–**h**), CO_2_ emission of crop rotations: Sunflower/spring treatments (**i**–**l**), and CO_2_ emission of crop rotations: triticale/spring treatments (**m**–**p**). Vertical bars represent the LSD of the t-test (p < 0.05)

The highest average of CO_2_ flux (0.76, 0.58, 0.48, and 0.29 g CO_2_ m^2^ days^−1^) were observed during the summer seasons of 2013/2014 and 2014/2015, and spring and fall-winter, respectively. Overall, the CO_2_ emission followed the behavior of average soil temperatures between crop seasons (28.9; 27.5; 25.1 and 21.1 °C), as well as soil moisture (0.138; 0.176 0.125, 0.141 m^3^ m^−3^), respectively, for the soybean 2013/2014 (Fig. 4a, e), soybean 2014/2015 (Fig. 4d, h), spring (Fig. 4c, g), and fall-winter seasons (Fig. 4b, f). Except for the soil moisture of the soybean 2013/2014, the CO_2_ emissions were associated with soil temperature (Fig. 4a, d) and humidity (Fig. 4e, h), with no distinctions between crop rotations.

CO_2_ emission peaks were observed in the first assessments after sowing their own crops in all seasons. In both soybean seasons (2013/2014 and 2014/2015), the fallow treatment was highlighted by variation in CO_2_ emissions. In the first assessments after soybean sowing (2013/2014), CO_2_ emissions from fallow at 1, 3 and 8 DAS were lower [(p < 0.05) 0.14; 0.16 and 0.12 g CO_2_ m^2^ h^−1^, respectively], compared to spring crops (averaged 0.51; 0.41 and 0.3 g CO_2_ m^2^ h^−1^, respectively). Interestingly, at 60 DAS of soybean (2013/2014), the highest average CO_2_ emission among crop rotations was observed (1.72 g CO_2_ m^2^ h^−1^), and the fallow treatment had the largest (p < 0.05) peak (average 2.4 g CO_2_ m^2^ h^−1^) independent of fall-winter crops. At 120 DAS, some CO_2_ emission peaks were observed through crop rotations sunflower/forage sorghum and triticale/sunn hemp (around 2 g CO_2_ m^2^ h^−1^).

The lowest CO_2_ emission (p < 0.05) by fallow treatment was also observed at 1, 3, and 8 DAS soybean 2014/2015 season (0.18; 0.18 and 0.5 g CO_2_ m^2^ h^−1^, respectively), compared to spring crops (average of 0.51; 0.49 and 0.8 g CO_2_ m^2^ h^−1^ respectively). Whereas at 60 DAS soybean, the fallow treatment when triticale was cropped in fall-winter had the highest CO_2_ emission (p < 0.05) compared to spring crop species (Fig. 4p). In general, for the other evaluations, there was no significant dispersion in CO_2_ emission between crop rotations.

### Cumulative and relative CO_2_ emissions

The average cumulative emissions were higher in soybean seasons, mainly in 2013/2014, with 56% higher than soybean 2014/2015 season (Additional file 2: Table S2). Both soybean seasons accounted for 47% and 31% of the total cumulative C–CO_2_ emissions (16.3 Mg C–CO_2_ ha^−1^) in 2013/2014 and 2014/2015, respectively. While in fall-winter and spring crops comprised only 15% and 6% of total cumulative emissions, respectively.

In the spring season (p < 0.05), as well as the total cumulative value (Additional file 2: Table S2), the accumulated C–CO_2_ emissions varied according to the treatments. The fallow treatment resulted in the highest overall cumulative CO_2_ emission (p < 0.05), about 1.4 t C–CO_2_ ha^−1^ higher than the average for spring cover crops. This result was higher than even accumulated in the spring crop (1.05 Mg C–CO_2_ ha ^−1^). In the soybean 2013/2014 season, the CO_2_ emissions were 23% higher (p = 0.08) from fallow compared to the average cover crop species. However, it is noteworthy that during the spring season, when soil was uncovered (fallow treatment), the cumulative emission was 45% lower (p < 0.05) compared to the average from cover crop species.

Relative emission was also affected by spring treatments (p < 0.05). Throughout the soybean seasons, to achieve a similar soybean yield under the fallow rotation, there was 30% higher emission (4.43 kg C–CO_2_ kg^−1^ of grains), compared to the average cover crop species (Additional file 2: Table S2).

### Carbon balance

**Fig. 5:**
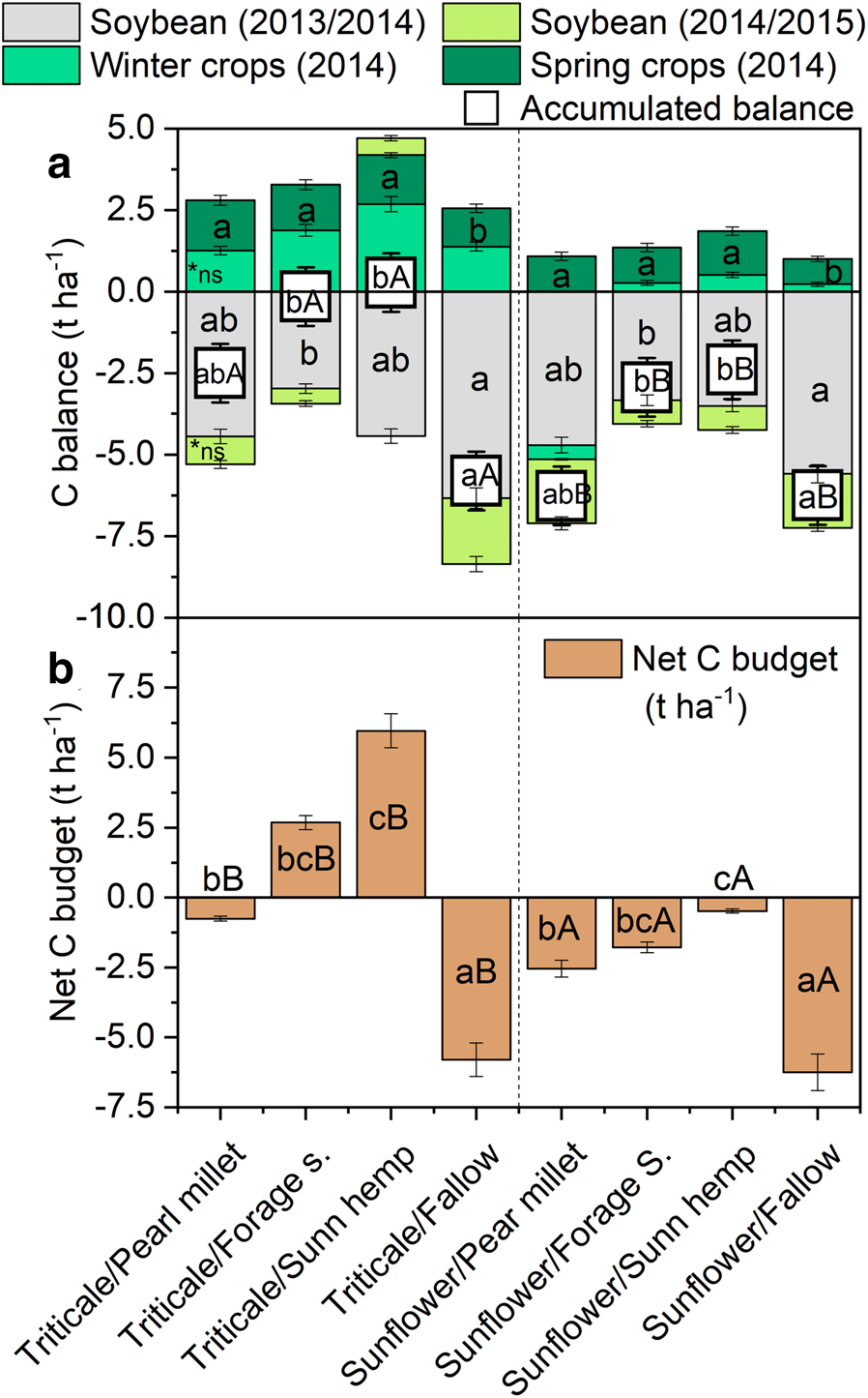
Carbon Balance in soybean 2013/2014, spring 2014, fall-winter 2014, and soybean 2014/2015 seasons, and the accumulate C balance (**a**), and Net C budget accounting the soil C stocks (**b**). Error bars represent standard deviation of data. *Means followed by different letters, lower-case letters within spring treatments and upper-case letters within winter crops differ from each other by the paired t-test (LSD, p < 0.05)

The C balances during spring and fall-winter crops were positive. However, in both soybean seasons, the C balances were negative (Fig. 5a). Considering the overall balances, it is possible to observe distinct behaviors between crop rotations at different levels. Triticale had a better C balance compared to sunflower (p < 0.05); i.e., no one crop rotation with sunflower resulted in a positive C balance. Among spring treatments, the fallow had a higher negative C balance (p < 0.05), about − 6 t C ha^−1^, followed by pearl millet [(p < 0.05), − 4.3 t C ha^−1^]. While C balances were similar (p < 0.05) under rotations with forage sorghum and sunn hemp as spring crops, changing to negative when sunflower was cropped (− 2.9 and 2.4 t C ha^−1^, respectively), closer to neutral with triticale (− 0.1 and 0.2 t C ha^−1^, respectively). When accounting for the differences between 2013 C stocks (0–0.1 m) between spring treatments, setting fallow as a reference, we observed a better Net C balance among all spring species (Fig. 5b). In addition, the C balance became positive in sunn hemp and forage sorghum when cropped after triticale (6.0 and 2.6 t C ha^−1^, respectively).

## Discussion

### C and N in crop residue and soil

The adequate choice of crop rotation species is essential for the conservation and sustainability of production systems, especially for the amount and quality of crop residue left in soil surface under NT [7]. This condition is even more pronounced in tropical regions, whereas maintaining the soil surface covered during the dry winter is a challenge [29]. Thus, the high amount and C input during the dry winter by triticale residue (Fig. 2), with high ratios of lignin/N and C/N (Fig. 3), allowed the soil cover, in addition to the grain yield, of around of 1.4 Mg ha^−1^ (data not shown). In contrast, cropping sunflower has been inefficient for grain yield production, as observed in previous years [14, 30], even though sunflowers are considered drought tolerant [31]. Typically, grass species have greater potential to produce biomass mainly under water deficit conditions and the residue composition with esterified acetylated lignin complexes [32], which implies slower decomposition. Besides the amount of crop residue on soil surface, it is important to highlight the quality of crop residues, characterized by biochemical composition and C/N ratios, which stimulates the mineralization or persistence on soil surface [7, 14, 30]. Therefore, C losses must be offset compensated by an increase in the amount of crop residues on the soil surface, either by cover crops as well as by cash crops.

The quality condition of crop residue to maintain soil covered during fall-winter is replaced by the high N inputs in the spring, the rain season, to stimulate residue mineralization. In this sense, the higher N input from sunn hemp is explained by it being among the leguminous species with the highest capacity to fix atmospheric N [33]. It is noteworthy that the N input from sunn hemp was almost twice as much as the fallow treatment, which is relevant in cropping systems with the absence of N fertilization. Also, according to the crop residue biochemical composition, sunn hemp is classified as high quality, or class I, different from other species in our soybean cropping systems considered medium quality, or class III [34].

Larger C and N inputs from sunn hemp and forage sorghum in spring, coupled with the high-quality residues of this legume, explain the larger C stock and SOC and TN contents on soil surface, especially with the legume cultivation compared to fallow in the spring. The crop residue characteristics are essential for increasing soil surface SOC, and TN retention under NT [35, 36], and confirm the importance of N supply for soil C sequestration [37]. Our results are in agreement with several studies in different soil and climatic conditions, in which the largest C inputs are associated with the largest soil C stocks [38, 39]. According to [38], cover crops in production systems are considered a key element in the reduction of C footprints due to the positive effect on SOC, being a potential CO_2_ mitigation strategy. Similarly, the limited SOC and TN content, generally observed at fallow, could be explained by the lower crop residue inputs (Fig. 2). According to [40] the intensification of cropping systems with the elimination of fallow periods under NT may restore SOC and improve soil structural properties. The fallow soil has lower microbial diversity, which is considered essential for the cycling of C and N nutrients [41]. Another feature is the fast mineralization of soybean residues in succession with the fallow treatment, which stimulates SOC loss rates and erosion [42].

### CO_2_ emissions

The climate conditions were typical of the region across the crop seasons, with typically mild temperatures and low rainfall during fall-winter (Fig. 1). The soil temperature (Tsoil) and moisture (Msoil) helps explain the lower CO_2_ emission during fall-winter (Fig. 4j, n), as well in the spring, the rainfall events associated with the increase of temperature and consequently the CO_2_ emission (Fig. 4). The CO_2_ emissions in agro-ecosystems is a result of the integration of crop-root respiration and microbial respiration, and soil C dynamics [43]. Tsoil and Msoil regulate the microbial activity and SOM mineralization [44, 45], and are recognized as the main factors driving soil CO2 fluxes [43].

Higher CO_2_ flux was expected at the beginning of soybean seasons, as it coincides with the high residue mineralization rates [14, 46, 47]. However, the lower rainfall events associated with high Tsoil, mainly in 2013 [(between December 2013 to January 2014), Fig. 1a], limited the initial CO_2_ fluxes. In tropical conditions, low Msoil associated with high Tsoil may restrict the flow of CO_2_, possibly due to moderate soil microbial activity [14]. Furthermore, with the increase in Msoil [(~ 0.2 m^3^ m^−3^), Fig. 4e], the CO_2_ emission increased (Fig. 4i, j). According to [48], microbial activity is reestablished with soil rewetting, increasing CO_2_ flux, and substrate mineralization.

Among the crop rotations, spring treatments were conditioning factors in CO_2_ fluxes across the seasons, when we analyzed the sunflower and triticale. In the firsts assessments in both soybean seasons (Fig. 4i, l, m, p), lower CO_2_ emissions were observed by fallow treatment compared to cover crops, as already expected [49], due to the absence of substrate. Hence, crop residues left on soil surface influences CO_2_ dynamics under NT. Comparing our results with emission from other studies, it is noted that the CO_2_ emission in soybean seasons from our study were similar to the soybean under NT in a gray Luvisol with crop residue on soil surface [50]. In an Oxisol with soybean-maize rotations under NT with winter crops (1.29 to 1.53 Mg ha^−1^ of residue), a slight lower CO_2_ flux were observed [51]. According to the authors the crop residue affects soil temperature and moisture and consequently the CO_2_ emission. The CO_2_ emission differences could be attributed to the climate conditions, especially soil moisture and temperature, and microbial activity, as well as the crop species, soil type, and its management. Cropping systems increase the microbial community dynamics compared to bare fallow soil [52]. The contribution of labile C from cover crop residue and their quality are the main factors influencing the microbial biomass and community structure [53, 54], and according to the mineralization rate may stimulate the CO_2_ flux [14].

Interestingly, CO_2_ emission peaks of fallow treatment differed from crop species (p < 0.05), especially at 60 DAS soybean (2013/2014). The breakdown of aggregates and exposure of previously protected C to microbial activity [3], combined with chiseling (spring 2013), stimulates CO_2_ emissions with soil rewetting and consequently, the microbial activity [55]. Probably the fallow management leads to high soil respiration on arable lands [56], at least with soil moisture re-establishment.

### Cumulative and relative CO_2_ emission

Root respiration contributes around 50% of total soil respiration [49], which helps explain the lower accumulated emission in spring by fallow treatment. However, when observing the impact throughout the crop seasons, there was a higher accumulated emission by fallow treatment. Typically, cropping cover crops improve the net C balance of the ecosystem by replacing fallow periods (carbon source) with an additional period of C assimilation [3, 57, 58]. Our results agree with a review that emphasizes the potential that cover crops have to mitigate climate change, especially sequestering C, reducing the inorganic fertilizer and soil erosion [10].

Our results demonstrate that cover crops increased soybean production efficiency, and the relative CO_2_ emission values were similar to other studies with this main crop under NT [14, 45, 50]. The cumulative emission in soybean (2014/2015 season) from our experiment (on averaged of 4.9 Mg C–CO_2_ ha^−1^) was extremely close to the estimation on soybean growing (on averaged of 4.5 Mg C–CO_2_ ha^−1^), cropping the rotation wheat-soybean under NT in a Gray Luvisol [50]. Similarly, the authors observed a greater CO_2_ emission in treatments with crop residue left in soil surface than under no residue plots. In addition, also a lower relative CO_2_ emission per unit soybean grain. Likewise, although the crop residues increased CO_2_ emission, the soybean yield was enhanced, as also observed by [59] in rice in a Gleyic Luvisol. Therefore, the conservation agriculture could improve resource-use efficiency in terms of crop yield according to crop rotations. According to [60], enhancing agricultural productivity per unit area is a viable option for mitigating GHG emissions. However, the sustainable management of natural resources with the intensification of agricultural production is also essential.

Even though the fallow treatment momentarily resulted in lower CO_2_ emissions, throughout the crop seasons, higher accumulated emissions reduced the soybean production efficiency. For this reason, fallow-based crop successions, in the long-term tend to be less efficient [61]. This suggests that the inclusion of cover crops contribute significantly to mitigating CO_2_ emissions.

### C balance

Several studies have also observed a negative C balance in soybean cropping systems [56, 62–64], acting as a C source rather than a sink. Between both soybean crop seasons, the negative balance in 2013/2014 stands out, associated with the water deficit related to high temperature. These climatic conditions limit the crop yields and hence, the C balance [64]. In the next decades, according to climate change projections, these events could be more typical [2]. Allied to the fact that cropping soybean has lower C accumulation than maize, for example, mainly under water stress and temperature increase [64]. Hence, the efficiency of cropping production systems should be considered in a long-term crop rotations.

The grain yield and C exportation from triticale (average 0.64 Mg ha^−1^, data not shown), makes it possible to equalize the C balance and its balance in crop rotations with the cereal, mainly when cropped before sunn hemp or forage sorghum in the spring season, confirming partially our hypothesis. On the other hand, the typical water deficit on fall-winter limits the sunflower yield, making the C negative balance independent of spring crop rotations.

Negative C balance from bare soil/fallow in succession to soybean crop was also observed [63]. On the other hand, cropping in spring season is essential to increase SOC, TN, as seen in previous years, and C stocks at 0–0.1 m soil depth, as well as improving the C balance of soybean cropping systems under NT. According to [65], the C balance is favored according to the amount of crop residues on soil surface, allowing the agricultural systems to act as CO_2_ sink. Therefore, the CO_2_ mitigation may be an additional ecosystem service provided by a few cover crops in rotation in tropical conditions.

## Conclusion

Long-term studies are essential for better understanding crop influences on C-scale ecosystem dynamics. The C and N inputs from crop residues left on soil surface under NT influenced the CO_2_ emissions, SOC and TN, and C stocks at 0–0.1 m depth, the relative soybean emission, and C balance. In general, the largest C and N inputs from sunn hemp or forage sorghum in spring season increased SOC and C stocks, and resulted in positive C balances when cropped in succession to triticale in fall-winter.

Our results indicate that the crop rotation species can contribute to mitigating the CO_2_ emissions, both by increasing the C input from crop residues as well as C sequestration. The adequate crop rotation species makes the soybean cropping systems more efficient, with a positive C balance. Thus, the crop rotation species in tropical conditions comprising an important tool in mitigating global warming potential. Our data provide support for further studies about crop sequence to assess the C sequestration. Additional impacts on C balance are hypothesized, assuming specifically the results from the legume in cover crops following the maize as the main crop in rotation.

## Supplementary information

**Additional file 1: Table S1.** Crop sequences used in the experiment since 2003.

**Additional file 2: Table S2.** TN, SOC, and stocks at 0-0.1 m, relative and cumulative C-CO_2_ emissions.

## Data Availability

Data is available in Springer nature

## Abbreviations

CO_2_: Carbon dioxide
DAS: Days after sowing
GHG: Greenhouse gas
C: Carbon
N: Nitrogen
NT: No-till
SD: Soil bulk density
SL: Soil layer
SOC: Soil organic carbon
TN: Total soil nitrogen

## References

[1] Farina R, Marchetti A, Francaviglia R, Napoli R, Di Bene C (2017). Modeling regional soil C stocks and CO_2_ emissions under Mediterranean cropping systems and soil types. Agric Ecosyst Environ..

[2] Davidson EA, Janssens IA (2006). Temperature sensitivity of soil carbon decomposition and feedbacks to climate change. Nature.

[3] Lal R (2002). The potential of soils of the tropics to sequester carbon and mitigate the greenhouse effect. Adv Agron.

[4] Lal R (2004). Soil carbon sequestration impacts on global climate change and food security. Science (80-).

[5] Carbon Brief. The carbon brief profile: Brazil. 2018. https://www.carbonbrief.org/the-carbon-brief-profile-brazil. Accessed 05 Sept 2019.

[6] Powlson DS, Stirling CM, Thierfelder C, White RP, Jat ML (2016). Does conservation agriculture deliver climate change mitigation through soil carbon sequestration in tropical agro-ecosystems?. Agric Ecosyst Environ.

[7] Rigon JPG, Franzluebbers AJ, Calonego JC (2020). Soil aggregation and potential carbon and nitrogen mineralization with cover crops under tropical no-till. J Soil Water Conserv..

[8] Gonzalez-Sanchez EJ, Veroz-Gonzalez O, Blanco-Roldan GL, Marquez-Garcia F, Carbonell-Bojollo R (2015). A renewed view of conservation agriculture and its evolution over the last decade in Spain. Soil Tillage Res..

[9] Basche AD, Archontoulis SV, Kaspar TC, Jaynes DB, Parkin TB, Miguez FE (2016). Simulating long-term impacts of cover crops and climate change on crop production and environmental outcomes in the Midwestern United States. Agric Ecosyst Environ.

[10] Kaye JP, Quemada M (2017). Using cover crops to mitigate and adapt to climate change. A review. Agron Sustain Dev..

[11] Kögel-Knabner I (2002). The macromolecular organic composition of plant and microbial residues as inputs to soil organic matter: fourteen years on. Soil Biol Biochem.

[12] Paul BK, Vanlauwe B, Ayuke F, Gassner A, Hoogmoed M, Hurisso TT (2013). Medium-term impact of tillage and residue management on soil aggregate stability, soil carbon and crop productivity. Agric Ecosyst Environ.

[13] Hiel MP, Barbieux S, Pierreux J, Olivier C, Lobet G, Roisin C (2018). Impact of crop residue management on crop production and soil chemistry after seven years of crop rotation in temperate climate, loamy soils. PeerJ..

[14] Rigon JPG, Calonego JC, Rosolem CA, Scala NL (2018). Cover crop rotations in no-till system: short-term CO2 emissions and soybean yield. Sci Agric.

[15] de Araújo Santos GA, Moitinho MR, de Oliveira Silva B, Xavier CV, Teixeira DDB, Corá JE (2019). Effects of long-term no-tillage systems with different succession cropping strategies on the variation of soil CO2 emission. Sci Total Environ.

[16] Xavier CV, Moitinho MR, De Bortoli Teixeira D, André de Araújo Santos G, de Andrade Barbosa M, Bastos Pereira Milori DM (2019). Crop rotation and succession in a no-tillage system: implications for CO2 emission and soil attributes. J Environ Manage..

[17] Gentile R, Vanlauwe B, Chivenge P, Six J (2008). Interactive effects from combining fertilizer and organic residue inputs on nitrogen transformations. Soil Biol Biochem.

[18] Cotrufo MF, Wallenstein MD, Boot CM, Denef K, Paul E (2013). The Microbial Efficiency-Matrix Stabilization (MEMS) framework integrates plant litter decomposition with soil organic matter stabilization: do labile plant inputs form stable soil organic matter?. Glob Chang Biol..

[19] Bayer C, Gomes J, Zanatta JA, Vieira FCB, Dieckow J (2016). Mitigating greenhouse gas emissions from a subtropical Ultisol by using long-term no-tillage in combination with legume cover crops. Soil Tillage Res..

[20] Wang W, Akhtar K, Ren G, Yang G, Feng Y, Yuan L (2019). Impact of straw management on seasonal soil carbon dioxide emissions, soil water content, and temperature in a semi-arid region of China. Sci Total Environ.

[21] Pimentel LG, Weiler DA, Pedroso GM, Bayer C (2015). Soil N_2_O emissions following cover-crop residues application under two soil moisture conditions. J Plant Nutr Soil Sci.

[22] Raij BV, Andrade JC, Cantarella H, Quaggio JA (2001). Chemical analysis for evaluation of the fertility of tropical soils.

[23] Smith KA, Mullins EC (1991). Soil analysis: physical methods.

[24] Gee G, Bauder JW, Klute A (1986). Particle-size analysis. Methods soil anal mineral method.

[25] Embrapa (1997). Manual De métodos De Análises De Solo.

[26] Veldkamp E (1994). Organic Carbon turnover in Three tropical Soils under Pasture after Deforestation. Soil Sci Soc Am J.

[27] Redin M, Recous S, Aita C, Chaves B, Pfeifer IC, Bastos LM (2018). Root and shoot contribution to carbon and nitrogen inputs in the topsoil layer in no-tillage crop systems under subtropical conditions. Rev Bras Ciência do Solo..

[28] Inc SI (2009). The SAS System for Windows.

[29] Jantalia CP, Dos Santos HP, Urquiaga S, Boddey RM, Alves BJR (2008). Fluxes of nitrous oxide from soil under different crop rotations and tillage systems in the South of Brazil. Nutr Cycl Agroecosystems..

[30] Raphael JPA, Calonego JC, Milori DMBP, Rosolem CA (2016). Soil organic matter in crop rotations under no-till. Soil Tillage Res..

[31] Hussain M, Farooq S, Hasan W, Ul-Allah S, Tanveer M, Farooq M (2018). Drought stress in sunflower: physiological effects and its management through breeding and agronomic alternatives. Agric Water Manag.

[32] del Río JC, Gutiérrez A, Rodríguez IM, Ibarra D, Martínez ÁT (2007). Composition of non-woody plant lignins and cinnamic acids by Py-GC/MS, Py/TMAH and FT-IR. J Anal Appl Pyrolysis..

[33] Chikowo R, Mapfumo P, Nyamugafata P, Giller KE (2004). Mineral N dynamics, leaching and nitrous oxide losses under maize following two-year improved fallows on a sandy loam soil in Zimbabwe. Plant Soil.

[34] Palm CA, Gachengo CN, Delve RJ, Cadisch G, Giller KE (2001). Organic inputs for soil fertility management in tropical agroecosystems: application of an organic resource database. Agric Ecosyst Environ.

[35] Frasier I, Noellemeyer E, Figuerola E, Erijman L, Permingeat H, Quiroga A (2016). High quality residues from cover crops favor changes in microbial community and enhance C and N sequestration. Glob Ecol Conserv..

[36] Yang Y, Huang Q, Yu H, Song K, Ma J, Xu H (2018). Winter tillage with the incorporation of stubble reduces the net global warming potential and greenhouse gas intensity of double-cropping rice fields. Soil Tillage Res..

[37] Van Groenigen JW, Van Kessel C, Hungate BA, Oenema O, Powlson DS, Van Groenigen KJ (2017). Sequestering soil organic carbon: a nitrogen Dilemma. Environ Sci Technol.

[38] Plaza-Bonilla D, Álvaro-Fuentes J, Bareche J, Pareja-Sánchez E, Justes É, Cantero-Martínez C (2018). No-tillage reduces long-term yield-scaled soil nitrous oxide emissions in rainfed Mediterranean agroecosystems: a field and modelling approach. Agric Ecosyst Environ.

[39] Lal R (2015). Sequestering carbon and increasing productivity by conservation agriculture. J Soil Water Conserv.

[40] Blanco-Canqui H, Holman JD, Schlegel AJ, Tatarko J, Shaver TM (2013). Replacing fallow with cover crops in a semiarid soil: effects on soil properties. Soil Sci Soc Am J.

[41] Novelli LE, Caviglia OP, Piñeiro G (2017). Increased cropping intensity improves crop residue inputs to the soil and aggregate-associated soil organic carbon stocks. Soil Tillage Res..

[42] Novelli LE, Caviglia OP, Melchiori RJM (2011). Impact of soybean cropping frequency on soil carbon storage in Mollisols and Vertisols. Geoderma.

[43] Sugihara S, Funakawa S, Kilasara M, Kosaki T (2012). Effects of land management on CO2 flux and soil C stock in two Tanzanian croplands with contrasting soil texture. Soil Biol Biochem.

[44] Khalil MI, Baggs EM (2005). CH4 oxidation and N2O emissions at varied soil water-filled pore spaces and headspace CH4 concentrations. Soil Biol Biochem.

[45] Zhang Z, Liang S, Wang J, Zhang X, Mahamood M, Yu J (2018). Tillage and crop succession effects on soil microbial metabolic activity and carbon utilization in a clay loam soil. Eur J Soil Biol..

[46] Gomes J, Bayer C, de Souza Costa F, de Cássia Piccolo M, Zanatta JA, Vieira FCB (2009). Soil nitrous oxide emissions in long-term cover crops-based rotations under subtropical climate. Soil Tillage Res..

[47] Peyrard C, Mary B, Perrin P, Véricel G, Gréhan E, Justes E (2016). N2O emissions of low input cropping systems as affected by legume and cover crops use. Agric Ecosyst Environ.

[48] Butterly CR, Marschner P, McNeill AM, Baldock JA (2010). Rewetting CO2 pulses in Australian agricultural soils and the influence of soil properties. Biol Fertil Soils.

[49] Oertel C, Matschullat J, Zurba K, Zimmermann F, Erasmi S (2016). Greenhouse gas emissions from soils—a review. Chemie der Erde Geochem.

[50] Langeroodi A, Reza S, Osipitan A, Radicetti E (2019). Benefits of sustainable management practices on mitigating greenhouse gas emissions in soybean crop (Glycine max). Sci Total Environ..

[51] Xavier CV, Moitinho MR, Teixeira DDB, de Araújo Santos GA, Corá JE, La Scala N (2020). Crop rotation and sequence effects on temporal variation of CO2 emissions after long-term no-till application. Sci Total Environ..

[52] Wienhold BJ, Pikul JL, Liebig MA, Mikha MM, Varvel GE, Doran JW (2006). Cropping system effects on soil quality in the Great Plains: synthesis from a regional project. Renew Agric Food Syst..

[53] Brennan EB, Acosta-Martinez V (2017). Cover cropping frequency is the main driver of soil microbial changes during six years of organic vegetable production. Soil Biol Biochem.

[54] Fanin N, Bertrand I (2016). Aboveground litter quality is a better predictor than belowground microbial communities when estimating carbon mineralization along a land-use gradient. Soil Biol Biochem.

[55] Haney RL, Hons FM, Sanderson MA, Franzluebbers AJ (2001). A rapid procedure for estimating nitrogen mineralization in manured soil. Biol Fertil Soils.

[56] Hernandez-Ramirez G, Hatfield JL, Parkin TB, Sauer TJ, Prueger JH (2011). Carbon dioxide fluxes in corn-soybean rotation in the midwestern US: Inter- and intra-annual variations, and biophysical controls. Agric For Meteorol..

[57] Nielsen DC, Calderón FJ, Hatfield JL, Sauer TJ. Fallow effects on soil; 2011.

[58] Duiker SW, Lal R (1999). Crop residue and tillage effects on carbon sequestration in a Luvisol in central Ohio. Soil Tillage Res..

[59] Dossou-Yovo ER, Brüggemann N, Jesse N, Huat J, Ago EE, Agbossou EK (2016). Reducing soil CO2 emission and improving upland rice yield with no-tillage, straw mulch and nitrogen fertilization in northern Benin. Soil Tillage Res..

[60] Popp A, Dietrich JP, Lotze-Campen H, Klein D, Bauer N, Krause M (2011). The economic potential of bioenergy for climate change mitigation with special attention given to implications for the land system. Environ Res Lett..

[61] Plaza-Bonilla D, Nolot JM, Raffaillac D, Justes E (2017). Innovative cropping systems to reduce N inputs and maintain wheat yields by inserting grain legumes and cover crops in southwestern France. Eur J Agron.

[62] Hollinger SE, Bernacchi CJ, Meyers TP (2005). Carbon budget of mature no-till ecosystem in North Central Region of the United States. Agric For Meteorol.

[63] Lewczuk NA, Posse G, Richter K, Achkar A (2017). CO2 and N2O flux balance on soybean fields during growth and fallow periods in the Argentine Pampas—a study case. Soil Tillage Res..

[64] Dold C, Büyükcangaz H, Rondinelli W, Prueger JH, Sauer TJ, Hatfield JL (2017). Long-term carbon uptake of agro-ecosystems in the Midwest. Agric For Meteorol.

[65] Duiker SW, Lal R (2000). Carbon budget study using CO2 flux measurements from a no till system in central Ohio. Soil Tillage Res..

