# Supplementary material for: Soil carbon fluxes and balances of crop rotations under long-term no-till
Source: Carbon Balance Manag. 2020 Sep 16;15:19. doi: 10.1186/s13021-020-00154-3 (PMC7493943; doi:10.1186/s13021-020-00154-3)
Supplement: Supplementary file 1 — Additional file 1: Table S1. Crop sequences used in the experiment since 2003. [file 13021_2020_154_MOESM1_ESM.docx]

**Table S1.** Crop sequences used in the experiment since 2003.

| year | crops seasons | treatments (crop sequences) | | | | | | | |
| --- | --- | --- | --- | --- | --- | --- | --- | --- | --- |
|  |  | 1 | 2 | 3 | 4 | 5 | 6 | 7 | 8 |
| 2003 | *^§^fall-winter* | *Sunfl. | Sunfl. | Sunfl. | Sunfl. | Tritic. | Tritic. | Tritic. | Tritic. |
|  | *^†^spring* | P. millet | F. sorgh. | Sunn h. | Fallow | P. millet | F. sorgh. | Sunn h. | Fallow |
|  | *^‡^summer* | -----------------------------------------------Soybean-------------------------------------------------- | | | | | | | |
| 2004 | *fall-winter* | Sunfl. | Sunfl. | Sunfl. | Sunfl. | Tritic. | Tritic. | Tritic. | Tritic. |
|  | *spring* | P. millet | F. sorgh. | Sunn h. | Fallow | P. millet | F. sorgh. | Sunn h. | Fallow |
|  | *summer* | -----------------------------------------------Soybean-------------------------------------------------- | | | | | | | |
| 2005 | *fall-winter* | Sunfl. | Sunfl. | Sunfl. | Sunfl. | Tritic. | Tritic. | Tritic. | Tritic. |
|  | *spring* | P. millet | F. sorgh. | Sunn h. | Fallow | P. millet | F. sorgh. | Sunn h. | Fallow |
|  | *summer* | -----------------------------------------------Soybean-------------------------------------------------- | | | | | | | |
| 2006 | *fall-winter* | Sunfl. | Sunfl. | Sunfl. | Sunfl. | Tritic. | Tritic. | Tritic. | Tritic. |
|  | *spring* | P. millet | F. sorgh. | Sunn h. | Fallow | P. millet | F. sorgh. | Sunn h. | Fallow |
|  | *summer* | -----------------------------------------------Soybean-------------------------------------------------- | | | | | | | |
| 2007 | *fall-winter* | Sunfl. | Sunfl. | Sunfl. | Sunfl. | Tritic. | Tritic. | Tritic. | Tritic. |
|  | *spring* | P. millet | F. sorgh. | Sunn h. | Fallow | P. millet | F. sorgh. | Sunn h. | Fallow |
|  | *summer* | -----------------------------------------------Soybean-------------------------------------------------- | | | | | | | |
| 2008 | *fall-winter* | Sunfl. | Sunfl. | Sunfl. | Sunfl. | Tritic. | Tritic. | Tritic. | Tritic. |
|  | *spring* | P. millet | F. sorgh. | Sunn h. | Fallow | P. millet | F. sorgh. | Sunn h. | Fallow |
|  | *summer* | -----------------------------------------------Soybean-------------------------------------------------- | | | | | | | |
| 2009 | *fall-winter* | Sunfl. | Sunfl. | Sunfl. | Sunfl. | Tritic. | Tritic. | Tritic. | Tritic. |
|  | *spring* | P. millet | F. sorgh. | Sunn h. | Fallow | P. millet | F. sorgh. | Sunn h. | Fallow |
|  | *summer* | -----------------------------------------------Soybean-------------------------------------------------- | | | | | | | |
| 2010 | *fall-winter* | Sunfl. | Sunfl. | Sunfl. | Sunfl. | Tritic. | Tritic. | Tritic. | Tritic. |
|  | *spring* | P. millet | F. sorgh. | Sunn h. | Fallow | P. millet | F. sorgh. | Sunn h. | Fallow |
|  | *summer* | -----------------------------------------------Soybean-------------------------------------------------- | | | | | | | |
| 2011 | *fall-winter* | Sunfl. | Sunfl. | Sunfl. | Sunfl. | Tritic. | Tritic. | Tritic. | Tritic. |
|  | *spring* | P. millet | F. sorgh. | Sunn h. | Fallow | P. millet | F. sorgh. | Sunn h. | Fallow |
|  | *summer* | -----------------------------------------------Soybean-------------------------------------------------- | | | | | | | |
| 2012 | *fall-winter* | Sunfl. | Sunfl. | Sunfl. | Sunfl. | Tritic. | Tritic. | Tritic. | Tritic. |
|  | *spring* | P. millet | F. sorgh. | Sunn h. | Fallow | P. millet | F. sorgh. | Sunn h. | Fallow |
|  | *summer* | -----------------------------------------------Soybean-------------------------------------------------- | | | | | | | |
| 2013 | *fall-winter* | Sunfl. | Sunfl. | Sunfl. | Sunfl. | Tritic. | Tritic. | Tritic. | Tritic. |
|  | *spring* | P. millet | F. sorgh. | Sunn h. | Fallow | P. millet | F. sorgh. | Sunn h. | Fallow |
|  | *summer* | -----------------------------------------------Soybean-------------------------------------------------- | | | | | | | |
| 2014 | *fall-winter* | Sunfl. | Sunfl. | Sunfl. | Sunfl. | Tritic. | Tritic. | Tritic. | Tritic. |
|  | *spring* | P. millet | F. sorgh. | Sunn h. | Fallow | P. millet | F. sorgh. | Sunn h. | Fallow |
|  | *summer* | -----------------------------------------------Soybean-------------------------------------------------- | | | | | | | |
| 2015 | *fall-winter* | Sunfl. | Sunfl. | Sunfl. | Sunfl. | Tritic. | Tritic. | Tritic. | Tritic. |
|  | *spring* | P. millet | F. sorgh. | Sunn h. | Fallow | P. millet | F. sorgh. | Sunn h. | Fallow |
|  | *summer* | -----------------------------------------------Soybean-------------------------------------------------- | | | | | | | |

*§* Fall-winter crops grown from second half of april and first half may to august/september;

*†* spring crops grown from september to second half of november;

*‡* soybean grown from seconf half of november and fist half december to april.

* Sunfl. – Sunflower; F. sorgh – Forage sorghum; Tritic – Triticale; Sunn h. – Sunn hemp;
