# Supplementary material for: Soil carbon fluxes and balances of crop rotations under long-term no-till
Source: Carbon Balance Manag. 2020 Sep 16;15:19. doi: 10.1186/s13021-020-00154-3 (PMC7493943; doi:10.1186/s13021-020-00154-3)
Supplement: Supplementary file 2 — Additional file 2: Table S2. TN, SOC, and stocks at 0-0.1 m, relative and cumulative C-CO2 emissions. [file 13021_2020_154_MOESM2_ESM.docx]

**Table S2.** TN, SOC, and stocks at 0-0.1 m, relative and cumulative C-CO_2_ emissions.

| Fall-winter | TN^a^ | SOC^b^ | N | C | relative soybean C-CO_2_ emission^c^ | Soybean^d^ | Fall-winter^e^ | Spring^f^ | Soybean^g^ | total^h^ |
| --- | --- | --- | --- | --- | --- | --- | --- | --- | --- | --- |
|  | content | | stock 2013 | |  | 2013/2014 | 2014 | 2014 | 2014/2015 |  |
|  | (g kg^-1^) | | (t ha^-1^) | | (kg C-CO_2_ kg^-1^) | C-CO_2_ emission (t C-CO_2_ ha^-1^) | | | | |
| Triticale | 1.98 | 21.50 | 2.71 | 29.2 | 3.68 | 8.02 | 2.49 | 1.13 | 4.75 | 16.50 |
| Sunflower | 1.99 | 21.42 | 2.70 | 29.0 | 3.66 | 7.43 | 2.41 | 0.96 | 5.15 | 16.10 |
| LSD | 2.02 | 2.06 | 0.14 | 1.80 | 0.46 | 1.63 | 0.19 | 0.26 | 1.88 | 1.44 |
| Spring | | | | | | | | | | |
| Pearl millet | 1.97 a | 20.8 ab | 2.73 ab | 28.9 ab | 3.67 a | 7.95 | 2.79 | 1.10 b | 5.22 | 17.1 ab |
| F. sorghum | 1.99 ab | 22.0 bc | 2.65 ab | 29.3 ab | 3.34 a | 6.78 | 2.35 | 1.24 b | 4.80 | 15.1 a |
| Sunn hemp | 2.07 b | 22.6 c | 2.83 b | 30.9 b | 3.24 a | 7.13 | 2.30 | 1.07 b | 4.67 | 15.5 ab |
| Fallow | 1.92 a | 20.4 a | 2.56 a | 27.2 a | 4.43 b | 9.04 | 2.37 | 0.78 a | 5.12 | 17.3 b |
| LSD | 0.09 | 1.30 | 0.22 | 2.70 | 0.51 | 1.90 | 0.55 | 0.21 | 0.90 | 3.5 |
| Fall-winter (W) | 0.93 | 0.85 | 0.84 | 0.80 | 0.91 | 0.33 | 0.27 | 0.14 | 0.54 | 0.37 |
| Spring (S) | 0.01 | <0.01 | 0.04 | 0.04 | <0.01 | 0.08 | 0.11 | <0.01 | 0.54 | 0.04 |
| (W) Vs. (S) | 0.63 | 0.42 | 0.56 | 0.86 | 0.52 | 0.77 | 0.39 | 0.19 | 0.4 | 0.56 |

^a^Total Nitrogen; ^b^Soil Organic Carbon; ^c^average from seasons 2013/2014 and 2014/2015; ^d^Soybean season 2013/2014; ^e^Fall-winter crops 2014; ^f^Spring crops 2014; ^g^Soybean season 2014/2015; ^h^cumulative C-CO_2_ emission. *Means followed by different letters, differ from each other by the paired t-test (LSD, p < 0.05)
